# Random Spacing between Metal Tree Electrodeposits in Linear DLA Arrays

**DOI:** 10.3390/e20090643

**Published:** 2018-08-28

**Authors:** Jaad Tannous, Lina Anouti, Rabih Sultan

**Affiliations:** 1 Department of Physics, American University of Beirut, P.O. Box 11-0236, Riad El Solh, 1107 2020 Beirut, Lebanon; 2 Department of Chemistry, American University of Beirut, P.O. Box 11-0236, Riad El Solh, 1107 2020 Beirut, Lebanon

**Keywords:** electrodeposition, fractals, inter-tree distance, entropy

## Abstract

When we examine the random growth of trees along a linear alley in a rural area, we wonder what governs the location of those trees, and hence the distance between adjacent ones. The same question arises when we observe the growth of metal electro-deposition trees along a linear cathode in a rectangular film of solution. We carry out different sets of experiments wherein zinc trees are grown by electrolysis from a linear graphite cathode in a 2D film of zinc sulfate solution toward a thick zinc metal anode. We measure the distance between adjacent trees, calculate the average for each set, and correlate the latter with probability and entropy. We also obtain a computational image of the grown trees as a function of parameters such as the cell size, number of particles, and sticking probability. The dependence of average distance on concentration is studied and assessed.

## 1. Introduction

Fractals are tree-like ramification structures that appear in a variety of physico-chemical systems [1]. They replicate on different length scales and are essentially self-similar [2,3]. Theoretical fractals are exact, and have a fixed fractal dimension [4], while natural or laboratory fractals are random and have a margin range for their fractal dimension. Typical examples of natural fractals with tree-like branching include lightning [5], rivers and tributaries [6], bacteria colonies [7,8], blood vessel and nerve networks [9], and, obviously, vegetation trees and shrubs [10,11].

In Chemistry, fractal structures are obtained essentially in precipitate [12,13] and metal electrodeposition systems [14,15]. Just as trees grow naturally along a linear array, trees of metal deposits can be grown by electrolysis or via a mere redox reaction. The former ones are known as electrodeposits, while the latter are coined *electroless* fractals.

**Figure 1:**
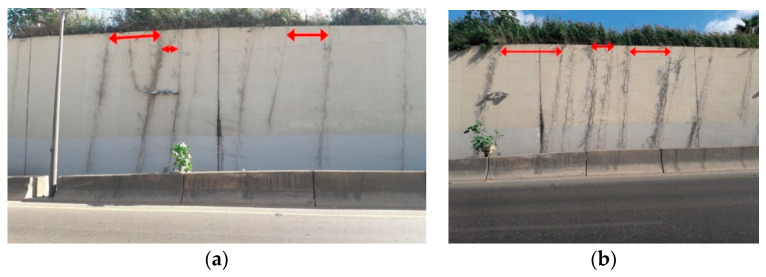
Spontaneous fractal plant growth pendent on the Beirut-Sidon highway wall (Lebanon). (**a**) November 2016. (**b**) September 2017. The red arrows reveal the variable inter-tree spacing.

The present study was motivated by the randomness observed in the location of plant stems grown naturally along a linear framework of soil imposed by a natural or artificial boundary. A typical illustrative example of such a phytosociological patterning scheme is depicted in Figure 1.

In forestry, the spatial patterns of tree locations have gained interest due to their role in understanding the historical and environmental topology of forests. Stem-mapping data characterizations [16] have shown that distances and gaps are important in delineating the mechanism of tree regeneration. Spatial analyses could explain the evolution of tree height, mortality, clustering, crown width, and crown class. Cottam and Curtis [17] used distance methods in phytosociological sampling to assess artificial random population in three natural forests. The distance between plants was viewed as an inverse measure of abundance and density. They considered the mean area of a trees cluster as the reciprocal of its density, with the distance and the square root of the area being directly correlated quantities. Markov chain Monte-Carlo algorithms [18], based on likelihood parameters, demonstrated means of predicting the phylogenetic evolution of a cluster of trees, with assessment of the most probable or “best” tree. Karp et al. [19] developed computational techniques to match repeated patterns in strings, trees and arrays. Repeated patterns and subtrees of a “full tree” are identified at various locations of root nodes.

In the present paper, we use distance measures to study randomness in the location of trees along a linear array of metal electrodeposits. Zinc (Zn) trees, grown by electrolysis and originating at a linear cathode, grow in a parallel regime and are known to exhibit fractal character [20]. Zn is deposited by reduction of Zn^2+^ from a ZnSO_4_ thin solution film, according to the scheme:

Zn^2+^ (*aq*) + 2e^−^ → Zn (*s*). (1)

We carry out four sets of experiments at seven (same) concentrations, wherein we measure the spacing between consecutive trees of height above a suitably chosen threshold. We observe random oscillations in the spacing sequence, and compute the entropy of this self-organizing system, which is shown to be consistent in all the considered sets. The present work is an extension of our previous work on electrolytic fractal growth in Zn [20], and two-metal [21,22] systems, and electroless growth of Ag by reduction of Ag^+^ with Cu [23].

## 2. Materials and Methods

**Figure 2:**
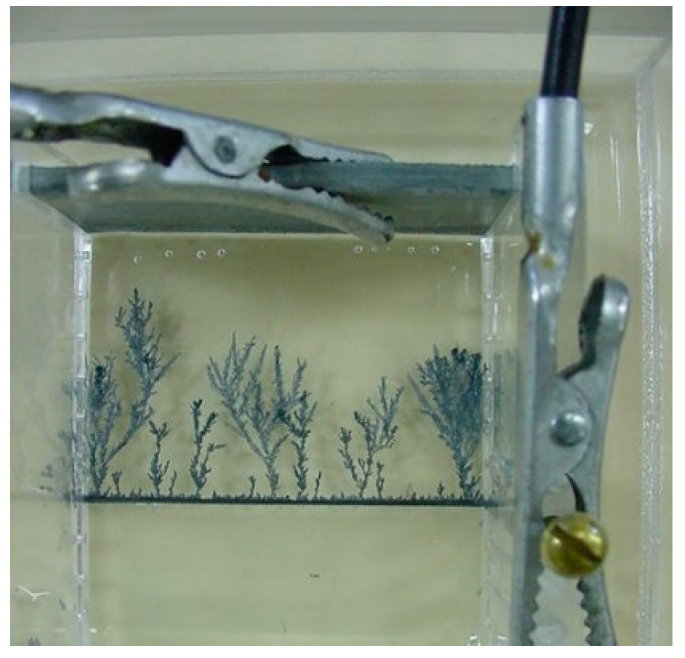
Experimental setup. The Zn fractal trees originate at the thin graphite cathode seen at the bottom, and grow toward the thick Zn anode. The applied voltage is 9.5 V.

A U-shaped methacrylate glass (plexiglass) cell is fitted with two electrodes: a thin cylindrical graphite cathode at the bottom (0.5 mm diameter), and a 3 mm thick Zn anode, as shown in Figure 2.

The cell is placed in a Petri dish containing a thin film (1.5 mm) of ZnSO_4_ solution of the desired concentration. The cell has a width of 5.0 cm and an electrode separation set at 4.0 cm. The electrodes are connected to a power supply [LG, graphite (−) and Zn (+)]. A potential difference of 9.5 V is turned on, marking the beginning of the electrolytic reaction (cathodic reduction according to Equation (1)).

**Figure 3:**
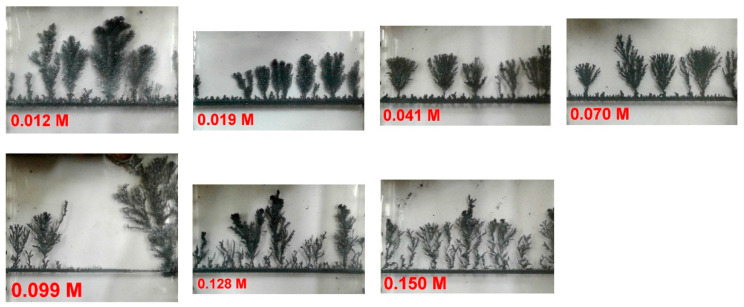
Fractal trees of Zn electrodeposits for experimental set No. 3, at seven different initial Zn^2+^ concentrations ([Zn^2+^]_0_), indicated on the corresponding frame. For other sets, similar tree-alley patterns are obtained, but of different distribution and textures.

In every run (seven different runs for each set corresponding to the concentrations 0.012, 0.019, 0.041, 0.070, 0.099, 0.128, and 0.150 M), the distances between consecutive trees of height ≥ 8% of the cell height span were measured by an image analysis technique. The obtained values were then averaged for each concentration. The electrolysis is left for a full span coverage in the cell (unless growth stopped before). Photographs of the obtained fractal patterns for Set No. 3 at the seven different concentrations are depicted in Figure 3.

## 3. Results

### 3.1. Random Oscillations

**Figure 4:**
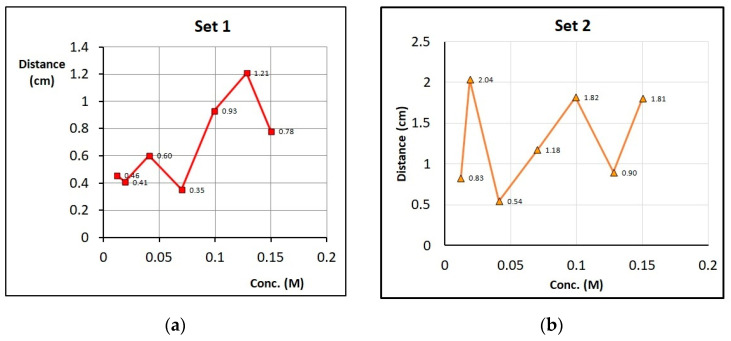

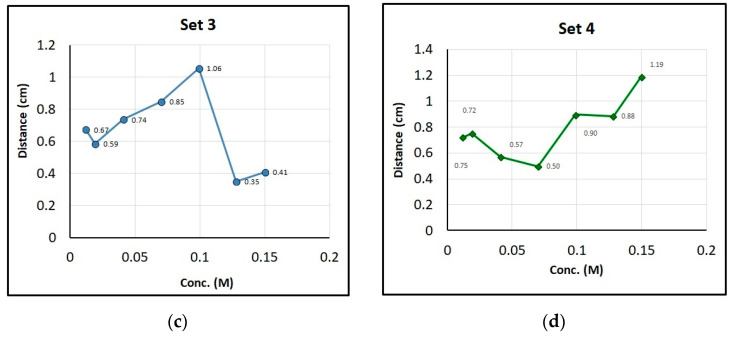
Plots of average inter-tree distance versus [Zn^2+^]_0_, for the **four** different sets. There are seven runs (concentrations) in each set. (**a**) Set 1. (**b**) Set 2. (**c**) Set 3. (**d**) Set 4.

Once the voltage (9.5 V) is set, Zn trees start growing at the cathode. The fractal electrodeposits (see Figure 3) have an average fractal dimension of 1.65 ± 0.05, consistent with the mechanism of Diffusion-Limited Aggregation (DLA). For each set (4 sets overall), the average distance between adjacent trees was plotted versus concentration. This average distance exhibits oscillations with concentration. The random oscillations for the four different sets are plotted in Figure 4.

### 3.2. Distance Entropy

To compute the entropy of a given set, we must assess the probability of a certain inter-tree spacing. We divided the range of spacings obtained into intervals of 0.20 cm. In a given set of seven concentrations, we calculated the probability of occurrence of a given distance falling within a specific range (0–0.20 cm; 0.20–0.40 cm; 0.40–0.60 cm, etc.) over all concentrations. The obtained probability *p_i_* of range numbered *i* is obtained by dividing the weight *n_i_* of range *i* (number of times it occurs) by the total number of measured inter-tree distances (*N*), in all runs at the different concentrations, as follows:

(2) $$p_{i}=n_{i}/N;\;N=\sum_{i}n_{i}$$

with this, the Shannon informational entropy [24] is given by:

(3) $$S=-\overset{N}{\underset{i=1}{\sum }}\;p_{i}\ln p_{i}$$

This formula has been used in different applications, notably to define the entropy of river networks [6]. In fractal ramification, the Shannon entropy has been associated with information fractal dimension [25], and was used for calculating the entropy of Liesegang patterns [26]. Although we are dealing with fractal systems, we do not adopt this approach here because we are focusing on the separation distances, and not the density of the ramification.

Table 1 lists the number *N* of outcomes (distance measurements) over all the concentrations for each of the four sets, along with the entropy *S* of each set calculated by Equation (3).

We notice that in spite of the great randomness revealed by the plots of Figure 4, and the erratic number of distances between “counted” trees in the second column of Table 1, the entropy maintains a nearly constant value of 1.78 ± 0.05, revealing the robustness of the characterization and the coherence of the system.

### 3.3. Theoretical Modeling

#### 3.3.1. Background

Fractal structures have been conjectured in both linear and circular geometry [14,27,28]. Molecular dynamics simulations were performed [29] to study the diffusion of Thouy and Jullien fractal aggregates as a function of their mass and fractal dimension. The diffusion coefficient of a fractal aggregate was found to vary linearly with the inverse of the simulation box length. Therefore, the intercept of a plot of *D* vs. *L*^−1^ is the diffusion coefficient corrected for finite size effects (infinite dilution limit). Fleury et al. [30] incorporated electro-convection and advection of ions in the fluid flow along with diffusion and electromigration, leading to a reduction in the size of the Chazalviel charge layer. The growth of zinc electrodeposits was studied experimentally in both linear [14,20] and circular [31] geometries, and simulated using a stochastic model based on the dielectric breakdown model (DBM). Using a mathematical model of coupled agglomeration and growth, Al_2_O_3_ monomer and agglomerated inclusion particles of hydrodynamic diameter between 6 μm and 9 μm were demonstrated [31] to accumulate, driven by a large swirl of flow in both inlet and outlet zones.

We now attempt to predict the concentration oscillations by proposing a model. To simulate the fractal patterns observed in electrodeposition, we employ the diffusion limited aggregation theory developed by Witten and Sander [32]. The modeling is done on MATLAB [33], adapting the algorithm described by Sagués and Costa [34]. The method is detailed in the next subsection.

#### 3.3.2. Method

The algorithm starts with a square grid, where particles are released from the top and diffuse through the grid via an isotropic random walk. The bottom array acts as the base of the aggregation sites, and a sticking probability to that base is assigned. Choosing eight nearest neighbors to a given particle, the latter moves toward the neighbor with highest probability according to a generated random number. Appropriate boundary conditions are obviously applied to make sure that the particle does not “wander off” the grid. The randomly walking particle sticks to an aggregated site if the probability is high enough. If the random number does not exceed the sticking probability, the particle will continue its motion until it aggregates. A variant of our algorithm from that developed by Sagués and Costa is that the latter authors used four nearest neighbors instead of eight (used here).

The algorithm takes 4 input variables, of which 2 are integers: the grid size and the number of pixels to be deposited. The other two input variables are generated randomly, and can take on real number values between 0 and 1. They are used for comparison with probabilities preset by the user. One of them probes the interaction between the pixels, while the other is for the interaction between the pixel and the bottom row.

The algorithm then proceeds to add pixels, one at a time, from the top of the grid. Each pixel undergoes a random walk until it either reaches an unoccupied cell with at least one occupied neighbor, or the bottom row of the grid. According to the 1 of the 2 generated random variables, the pixel sticks or does not stick. If the pixel sticks, the code saves the position then proceeds to add the next pixel from the top and the procedure is repeated. Otherwise, the pixel keeps diffusing until it sticks.

#### 3.3.3. Results

**Figure 5:**
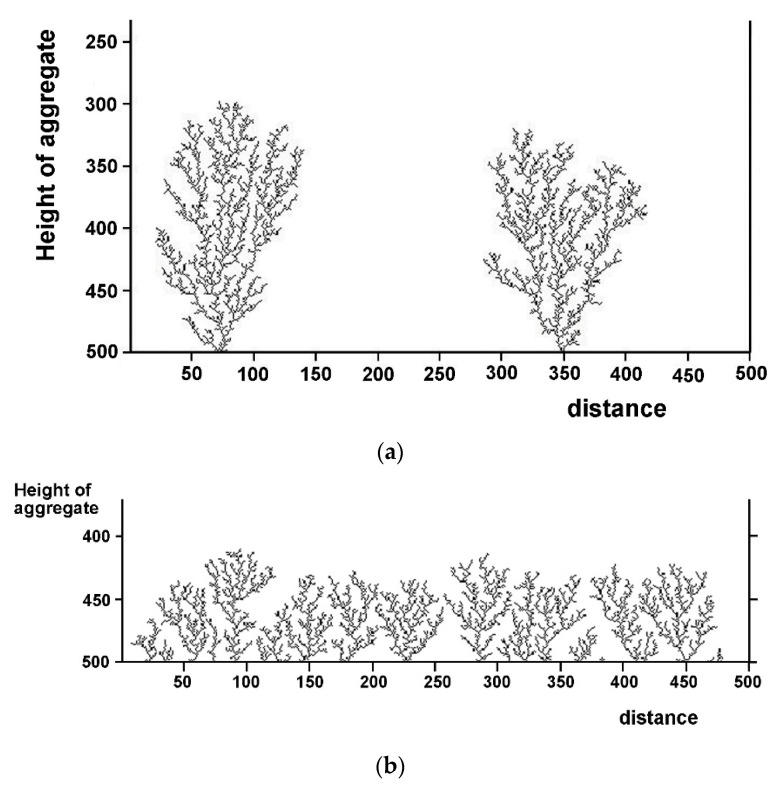
Simulated Diffusion-Limited Aggregation (DLA) trees originating at the bottom of a rectangular box (the cathode), computed by the method described in Section 3.3.2. (**a**) Total number of particles = 5000; particle on particle sticking probability = 0.6; particle on cathode sticking probability = 0.0001; (**b**) Total number of particles = 5000; particle on particle sticking probability = 0.6; particle on cathode sticking probability = 0.005.

Sample schemes of tree growth under two different choices of the model parameters (number of particles and sticking probability) are shown in Figure 5.

**Figure 6:**
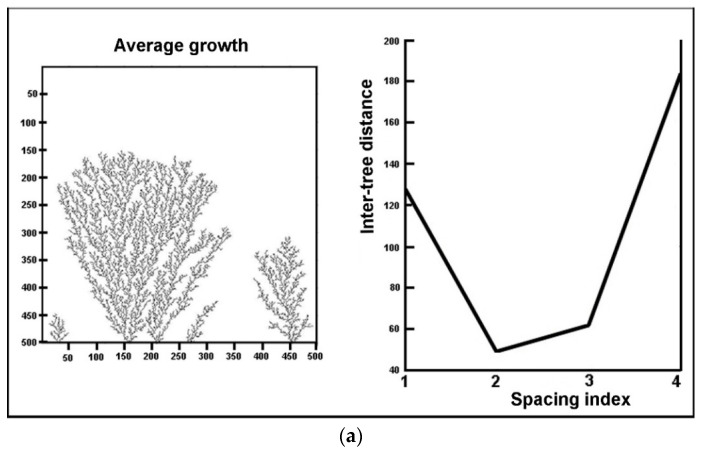

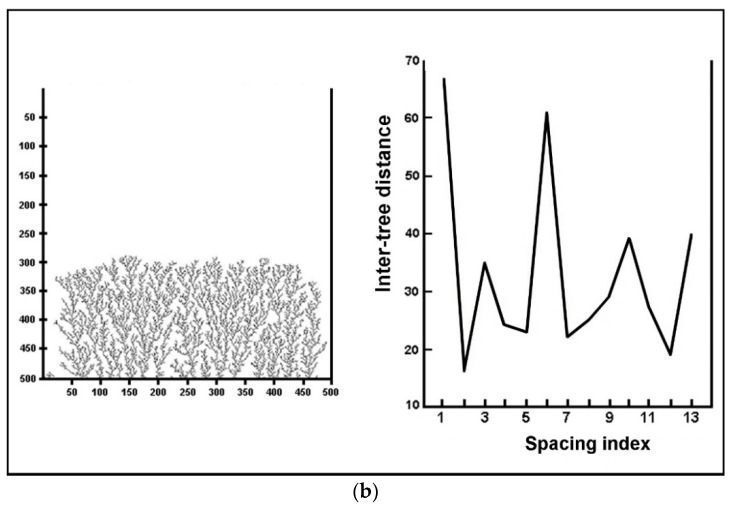
Simulated DLA trees originating at the cathode, along with the corresponding plot of inter-tree distance versus the spacing index between adjacent trees. (**a**) 15,000 pixels; sticking probability at cathode = 0.0002. (**b**) 15,000 pixels; sticking probability at cathode = 0.005.

We clearly see that the inter-tree distance is random, and hence an average distance between adjacent neighbors can be computed. To illustrate the random variation of spacing with tree number along the cathode (like on an alley of trees), we select two distinct concentrations and generate a plot of inter-tree spacing versus tree number (or spacing index). The concentration is characterized by the choice of the sticking probability (higher probability at higher concentration). The growth schemes and the corresponding plots are displayed in Figure 6 for two different conditions.

**Figure 7:**
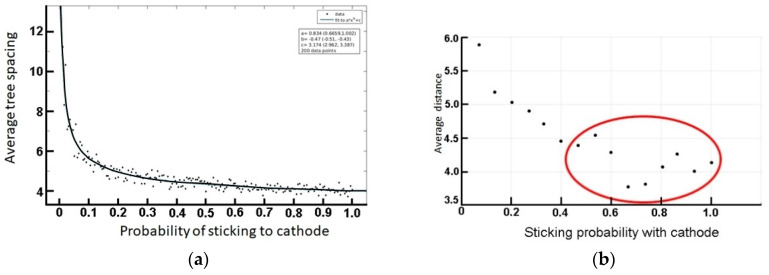
(**a**) A preliminary test for the variation of tree spacing with sticking probability. (**b**) Expanded scale to display a sample oscillation, in line with the results displayed in Figure 4.

Here again, we detect a great sensitivity on the initial parameters, which are themselves reminiscent of the experimental conditions. Finally, we study the variation of average spacing with concentration. A preliminary plot of average distance versus sticking probability (here equivalent to concentration) is depicted in Figure 7.

**Figure 8:**
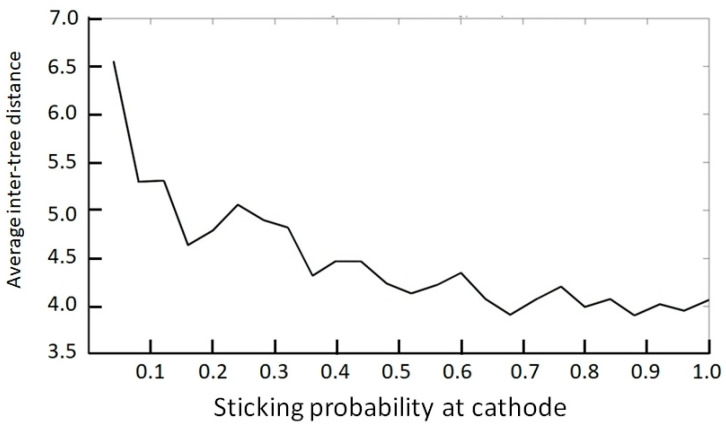
Variation of inter-tree distance with concentration or sticking probability, exhibiting random oscillations.

Although the overall trend is a net decrease (Figure 7a), the variation is essentially oscillatory. See notably the range highlighted inside the red oval of Figure 7b. The latter trend typically captures the obtained random variation portrayed in Figure 4. A closer look at the oscillatory behavior is highlighted in Figure 8, wherein the number of points (probability calculations) is decreased.

## 4. Conclusions

In conclusion, the random distance between growing trees is consistent over different sets of runs (each run is repeated at the same Zn^2+^ concentration within a given set). The tree locations are unpredictable and erratic, and hence no clear trend can be inferred. However, there is a rigorous coherence with the value of the entropy (1.78 ± 0.05), provided we use the same set of concentrations. The theoretical results capture the experimental findings in two major trends: randomness and distance oscillation with tree number within the same concentration, and random variation with concentration.

## Figures and Tables

**Table 1:** Number of inter-tree distances measured in each set of seven concentration runs, with the associated entropies (*S*).

Set	*N*	*S*
1	30	1.82
2	54	1.72
3	33	1.82
4	65	1.76

## References

[1] Ball P. (1999). The Self-Made Tapestry: Pattern Formation in Nature.

[2] Mandelbrot B.B. (1982). The Fractal Geometry of Nature.

[3] Viscek T. (1991). Fractal Growth Phenomena.

[4] Falconer K.J. (1988). The Hausdorff dimension of self-affine fractals. Math. Proc. Camb. Philos. Soc..

[5] Tsonis A.A., Elsner J.B. (1987). Fractal characterization and simulation of lightning. Beitr. Phys. Atmos..

[6] Claps P., Fiorentino M., Oliveto G. (1996). Informational entropy of fractal river networks. J. Hydrol..

[7] Matsuyama T., Matsushita M. (1993). Fractal morphogenesis by a bacterial cell population. Crit. Rev. Microbiol..

[8] Matsuyama T., Matsushita M. (1992). Self-similar colony morphogenesis by gram-negative rods as the experimental model of fractal growth by a cell population. Appl. Environ. Microbiol..

[9] Goldbeter A.L., Rigney D.R., West B.J. (1990). Chaos and fractals in human physiology. Sci. Am..

[10] Ball P. (2009). Branches, Nature’s Patterns: A Tapestry in Three Parts.

[11] Rudas A., Tόth I.P. (2013). Entropy and Hausdorff dimension in random growing trees. Stoch. Dyn..

[12] Toramaru A., Giochi A. (1996). Transition between periodic precipitation and tree-like crystal aggregates. J. Mineral. Soc. Jpn..

[13] Mandalian L., Sultan R. (2002). Fractal structures in PbF_2_/Pb(NO_3_)_2_ precipitate systems. Collect. Czechoslov. Chem. Commun..

[14] Matsushita M., Sano M., Hayakawa Y., Honjo H., Sawada Y. (1984). Fractal structures of zinc metal leaves grown by electrodeposition. Phys. Rev. Lett..

[15] Trigueros P.P., Claret J., Mas F., Sagués F. (1991). Pattern morphologies in zinc electrodeposition. J. Electroanal. Chem..

[16] Moeur M. (1993). Characterizing spatial patterns of trees using stem-mapped data. For. Sci..

[17] Cottam G., Curtis J.T. (1956). The use of distance measures in phytosociological sampling. Ecology.

[18] Larget B., Simon D.L. (1999). Markov chain Monte Carlo algorithms for the Bayesian analysis of phylogenetic trees. Mol. Biol. Evol..

[19] Karp R., Miller R., Rosenberg A. Rapid identification of repeated patterns in strings, trees and arrays. Proceedings of the 4th Annual ACM Symposium on Theory of Computing.

[20] Saab R., Sultan R. (2005). Density, fractal angle, and fractal dimension in linear Zn electrodeposition morphology. J. Non-Equilib. Thermodyn..

[21] Nakouzi E., Sultan R. (2011). Fractal structures in two-metal electrodeposition systems I: Pb and Zn. Chaos.

[22] Nakouzi E., Sultan R. (2012). Fractal structures in two-metal electrodeposition systems II: Cu and Zn. Chaos.

[23] Ibrahim H., Farah H., Zein Eddin A., Isber S., Sultan R. (2017). Ag fractal structures in electroless metal deposition systems with and without magnetic field. Chaos.

[24] Shannon C.E. (1948). A mathematical theory of communications. Bell Syst. Tech. J..

[25] Zmeskal O., Dzik P., Vesely M. (2013). Entropy of fractal systems. Comput. Math. Appl..

[26] Kalash L., Sultan R. (2014). Routes to fractality and entropy in Liesegang systems. Chaos.

[27] Grier D.G., Kessler D.A., Sander L.M. (1987). Stability of the dense radial morphology in diffusive pattern formation. Phys. Rev. Lett..

[28] Sawada Y., Dougherty A., Gollub J.P. (1986). Dendritic and fractal patterns in electrolytic metal deposits. Phys. Rev. Lett..

[29] Pranami G., Lamm M.H., Vigil R.D. (2010). Molecular dynamics simulation of fractal aggregate diffusion. Phys. Rev. E.

[30] Fleury V., Kaufman J., Hbbert B. (1993). Evolution of the space-charge layer during electrochemical deposition with convection. Phys. Rev. E.

[31] Chen C.P., Jorné J. (1990). Fractal analysis of zinc electrodeposition. J. Electrochem. Soc..

[32] Witten T.A., Sander L.M. (1981). Diffusion-limited aggregation, a kinetic critical phenomenon. Phys. Rev. Lett..

[33] MathWorks Inc. Matlab Computing Software.

[34] Sagués F., Costa J.M. (1989). A microcomputer simulation of fractal electrodeposition. J. Chem. Educ..

